# Comparative Evaluation of the Immune Responses in Cattle Mammary Tissues Naturally Infected with Bovine Parainfluenza Virus Type 3 and Bovine Alphaherpesvirus-1

**DOI:** 10.3390/pathogens8010026

**Published:** 2019-02-25

**Authors:** Selim ÇOMAKLI, Selçuk ÖZDEMİR

**Affiliations:** 1 Department of Pathology, Faculty of Veterinary Medicine, Atatürk University, Yakutiye 25240, Erzurum, Turkey; 2 Department of Genetics, Faculty of Veterinary Medicine, Atatürk University, Yakutiye 25240, Erzurum, Turkey; selcuk.ozdemir@atuni.edu.tr

**Keywords:** Bovine parainfluenza virus type 3 (BPIV-3), Bovine alphaherpesvirus-1 (BoHV-1), IFN-γ, CD4, CD8, mastitis

## Abstract

Bovine parainfluenza virus type 3 (BPIV-3) and Bovine alphaherpesvirus-1 (BoHV-1) lead to severe diseases in domesticated animals, such as Bovine, sheep, and goats. One of these diseases is mastitis, whose signs may not be observable in cases of viral infection due to the dominance of other clinical symptoms. This may lead to failure to predict viral agents in subclinical Bovine cases. Since viral infections have not been substantially investigated in mastitis studies, information about immune response to BPIV-3 and BoHV-1 infected Bovine mammary tissues may be inadequate. The present study aimed to determine the presence and prevalence of BPIV-3 and BoHV-1 agents in Bovine mammary tissues, and the immune response of such tissues against BPIV-3 and BoHV-1 infection. For this purpose, we first detected these viruses with qRT-PCR in mammary tissues. Then, we determined the expression profiles of interferon-γ (*IFN-γ*), *CD4*, and *CD8* genes with qRT-PCR. Lastly, we performed immunohistochemistry staining to identify the presence of *IFN-γ*, *CD4*, and *CD8* proteins in the mammary tissues. We found that 26, 16, and five of the 120 samples were BPI3-, BoHV1-, and BPIV-3 + BoHV-1 infected, respectively. Moreover, the gene expression levels of *IFN-γ* and *CD4* were strongly up-regulated in the virus-infected tissues, whereas the *CD8* gene expression level was only moderately up-regulated. Immunohistochemistry staining results were consistent with qRT-PCR results. Overall, our findings showed a high prevalence of BPIV-3 and BoHV-1 and indicated that cell-mediated immune response plays an important role against BPIV-3 and BoHV-1 infection in Bovine mammary tissues. Meanwhile, *IFN-γ* is an important cytokine for antiviral immunity against such infection.

## 1. Introduction

Mastitis is an inflammatory reaction of the parenchyma of the mammary gland. It can occur due to infectious, traumatic, or toxic agents and induce loss or reduction of milk yield [1,2]. In dairy animals, mastitis-causing pathogens are classified according to their epidemiological activity in infectious and environmental. Bacterial and non-bacterial pathogens, such as mycoplasms, fungi, yeasts, and chlamydia can lead to mastitis. Viruses take part in among the non-bacterial pathogens. Several viruses (including BoHV-1, BBovine herpes virus-4, foot-and-mouth disease virus, and BPIV-3) have been detected in milk from cows with clinical mastitis [3,4,5,6]. In addition, BoHV-1 and BPIV-3 have been observed in subclinical mastitis with no clinical findings [7].

The viral proteins are important for eliciting cellular immunity such as major glycoproteins, which play role in cell-mediated immunity [8] and for antibody-activated *CD4*+ T lymphocytes [9] or *CD8*+ cytotoxic lymphocytes [10,11]. *CD4*+ T helper (Th) cells serve as protective immune cells against intra-mammary infection as the major component of adaptive immune response. A previous study reported that mastitis caused the increasing of *CD4*+ cells in the milk of cattle [12]. *CD4*+ cells can secrete some cytokines, so they activate macrophages, *CD8*+ T cells, and B cells [13]. In mastitis, *CD4*+ T cells are mainly activated with recognition of complex molecules, which form between histocompatibility complex (MHC) II molecules or by antigen-presenting cells (APCs), B cells, and macrophages [13,14]. *CD8*+ T cells bind and clear off host cells, which are expressing foreign antigens related to MHC class I molecules. Suppressor *CD8*+ T cells play role in the immune response against bacterial infection [15].

Interferon-γ (*IFN*-*γ*) is the family of type II IFN and it is structurally not similar to type I IFNs, because it recognizes the different receptor, and is encoded by a different chromosomal locus. *CD4*+ cells, *CD8*+ cells, and NK cells particularly produce *IFN*-*γ* [16,17,18]. Besides, B cells, NKT cells, and professional APCs produce *IFN*-*γ* [19,20,21,22,23]. *IFN*-*γ* elevates the phagocytic capacity of neutrophils. Moreover, it was believed that *IFN*-*γ* is important in viral infections [24,25]. *IFN*-*γ* can lead to functional variation phagocytic cells in the mammary gland, thus it is an effective molecule in the control of Bovine mastitis [26,27].

The data obtained in the current study serves as a primary source for future studies of the pathogenesis of mastitis by determining the cellular immune responses to BoHV-1 and BPIV-3 in subclinical mastitis, which are manifest as single or mixed infections of cattle.

## 2. Results

### 2.1. Detection of Beta-Actin, BPIV-3, and BoHV-1 in Mammary Tissues

A ten-fold serial dilution of each of the total RNA was triplicate analyzed. All three targets were analyzed simultaneously and no evidence of cross reactivity between primers was detected. BPIV-3 agent was detected by the qRT-PCR in 26/120 samples, BoHV-1 agent in 16/120 samples. Coinfection with BPIV-3 and BoHV-1 was detected in 5/120 samples (Table 1). BPIV-3 and BoHV-1 infected tissues and their nucleic acid signals were shown in Figure S1. The expression satability of beta actin was demonstrated in Figure S2. In addition, the Ct/Cq values for BoHV-1 and BPIV-3 were given in the Table S1.

### 2.2. The Expression Profiles of IFN-γ, CD4, and CD8

**Figure 1:**
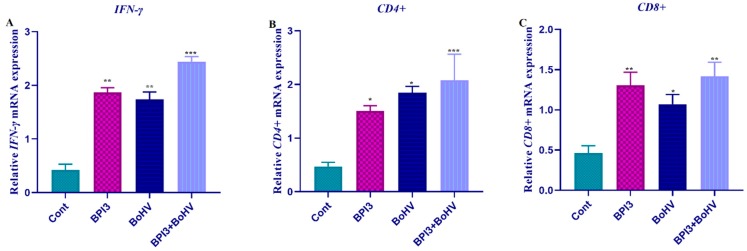
The mRNA transcript levels of *IFN-γ*, *CD4*, and *CD8* genes in natural BPIV-3 and BoHV-1 infected mammary tissues of cattle. Values represent the mean ± SD of three independent samples; error bars indicate standard deviation. Statistical significance (* *p* ˂ 0.05, ** *p* < 0.01 and *** *p* < 0.001) was analyzed using a one-way ANOVA. (**A**) Represent the relative mRNA expression levels of *IFN-γ* gene. (**B**) Represent the relative mRNA expression levels of *CD4* gene. (**C**) Represent the relative mRNA expression levels of *CD8* gene.

The expression profiles of *IFN*, *CD4*, and *CD8* genes were determined with qRT-PCR analysis. The mRNA transcript level of *IFN* was strongly up-regulated in the virus (BPIV-3, BoHV-1) infected samples compared to control (normal tissues). In the co-infected (BPIV-3 + BoHV-1) samples, *IFN* expression was similarly increased (Figure 1A). Similar to IFN gene expression profile, *CD4* mRNA transcript level was up-regulated in all infected samples compared to control (Figure 1B). In contrast, the expression profile of *CD8* was moderately up-regulated in all infected samples (Figure 1C). 

### 2.3. Immunohistochemistry Staining

**Figure 2:**
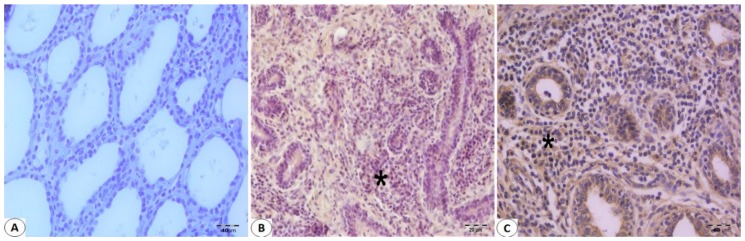
Comparison of positive and negative sections in terms of BPIV-3 and BoHV-1 virus in Bovine mammary tissue using paraffin-embedded sections (**A**–**C**): (**A**) Negative mammary section in terms of BPIV-3 and BoHV-1. Immunohistochemistry (IHC). Bar, 40 μm. (**B**) Positive T cell infiltration (star) in BPIV-3 infected mammary section. IHC. Bar, 20 μm. (**C**) Positive T cell infiltration (star) in BoHV-1 infected mammary section. IHC. Bar, 40 μm.

**Figure 3:**
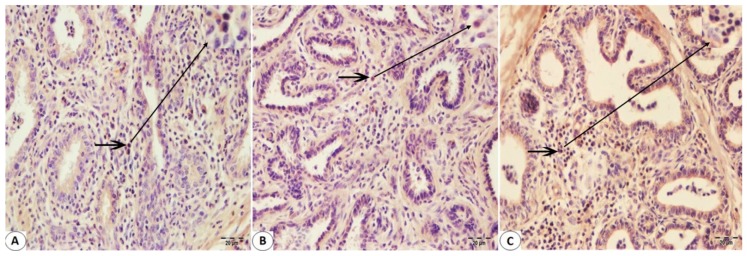
Immunohistochemical staining of CD8 + T cells of the Bovine mammary tissue using paraffin-embedded sections (**A**–**C**): (**A**) The intermediate immunoreactivity of CD8 + T lymphocytes (arrow) in the interalveolar connective tissue with the monoclonal antibody specific for CD8+ T cells in Bovine mammary tissue infected with BPIV-3. (**B**) The low immunoreactivity of T lymphocytes (arrow) in the interalveolar connective tissue with the monoclonal antibody specific for CD8+ T cells in Bovine mammary tissue infected with BoHV-1. (**C**) The high reactivity distributional of T lymphocytes in the interalveolar connective tissue with the monoclonal antibody specific for CD8+ T cells (arrow) in Bovine mammary tissue infected with BoHV-1 and BPIV-3. IHC. Bar, 20 μm.

**Figure 4:**
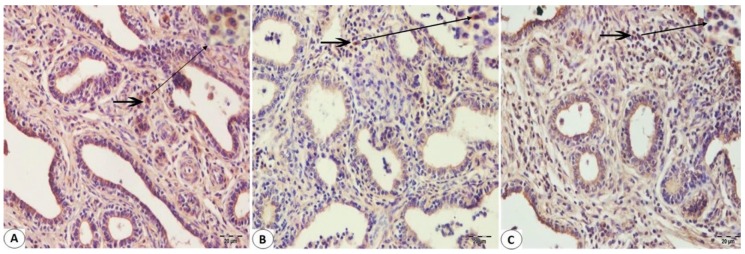
Immunohistochemical staining of CD4 + T cells of the Bovine mammary tissue using paraffin-embedded sections (**A**–**C**): (**A**) Showing the low immunoreactivity of T lymphocytes (arrow) in the interlobular connective tissue with the monoclonal antibody specific for CD4+ T cells in Bovine mammary tissue infected with BPIV-3. (**B**) The intermediate immunoreactivity of CD4 + T cells (arrow) in the interalveolar connective tissue with the monoclonal antibody specific for CD4+ T cells in Bovine mammary tissue infected with BoHV-1. (**C**) The high reactivity distributional of CD4 + T lymphocytes (arrow) in the interalveolar connective tissue with the monoclonal antibody specific for CD4+ T cells in Bovine mammary tissue infected with BoHV-1 and BPIV-3. IHC. Bar, 20 μm.

**Figure 5:**
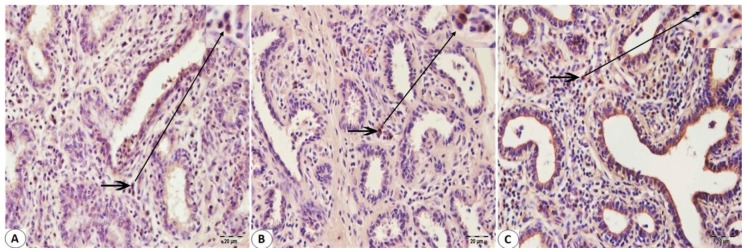
Immunohistochemical staining of IFN-γ in the Bovine mammary tissue using paraffin-embedded sections (**A**–**C**): (**A**) The intermediate immunoreactivity of IFN-γ (arrow) in the interalveolar connective tissue with the monoclonal antibody specific for IFN-γ in Bovine mammary tissue infected with BPIV-3. (**B**) The intermediate immunoreactivity of IFN-γ (arrow) in the interlobular connective tissue with the monoclonal antibody specific for IFN-γ in Bovine mammary tissue infected with BoHV-1. (**C**) The high immunoreactivity of IFN-γ (arrow) in the interalveolar connective tissue with the monoclonal antibody specific for IFN-γ in Bovine mammary tissue infected with BoHV-1 and BPIV-3. IHC. Bar, 20 μm.

Immunohistologically detectable immune cells were observed in Bovine mammary tissues infected with BPIV-3 and BoHV-1. No T cell infiltration was found in mammary sections not infected with Bohv-1 or BPIV-3 (Figure 2A). There was an increase in the number of T cells in the infected mammary sections with these viruses (Figure 2B,C). CD8+ T cell immunoreactivity was found to be greater than CD4+ T cell immunoreactivity in BPIV-3 infected Bovine mammary tissues (Figure 3A and Figure 4A). CD4+ T cell immunoreactivity was found to be greater than CD8+ T cell immunoreactivity in BoHV-1 infected Bovine mammary tissues (Figure 3B and Figure 4B). Besides, CD4+ T cell immunoreactivity was found to be greater than CD8+ T cell immunoreactivity in the co-infections which occurred by both agents (Figure 3C and Figure 4C). IFN-γ immunoreactivity was observed as moderate in both BPIV-3 and BoHV-1 infections (Figure 5A,B). However, IFN-γ immunoreactivity was observed to be too intense in the co-infections which occurred by BPIV-3 and BoHV-1 (Figure 5C). Immunoreactivity of CD4+, CD8+ T cell, and IFN-γ was observed in lymphocytes in the interlobular and interalveolar areas.

### 2.4. Comparative Results

There were significant differences between all groups in terms of CD4+, CD8+ T cell, and IFN-γ (*p* < 0.05, Table 2). CD4+ T cells immunoreactivity increased in co-infection status when compared to BPIV-3 or BoHV-1 infections (*p* < 0.05, Table 3). CD8+ T cell immunoreactivity increased BPIV-3 and co-infection status when compared to BoHV-1 (*p* < 0.05, Table 2). There was no difference for IFN-γ immunoreactivity among BoHV-1 and BPIV-3 infection according to co-infections (*p* > 0.05, Table 2).

## 3. Discussion

The current study provides information about the presence and distribution of viral agents BPIV-3 and BoHV-1, and *CD4*, *CD8*, and *IFN*-*γ* responses to natural BPIV-3 and BoHV-1 infection in the mammary tissues of cattle. We found that 26, 16, and five samples were BPIV-3, BoHV-1, and BPIV-3 + BoHV-1 infected, respectively. Both *CD4* and *IFN-γ* gene expressions were significantly up-regulated in the virus-infected tissues. In the co-infected (BPIV-3 + BoHV-1) tissues, the expression levels of *CD4* and *IFN-γ* gene were strongly increased. By contrast, the *CD8* gene expression level was only moderately increased in the virus-infected tissues relative to those of *CD4* and *IFN-γ*. These results were confirmed via immunohistochemistry staining. 

In qRT-PCR virus detection process, the accuracy of the data is ensured using positive control. However, positive control was not used in this study because of long process and biosafety precaution. In order to eliminate this limitation of the study, gel electrophoresis and melting curve analysis were performed. The consistency of the results indicates that the correct pcr products were detected. 

Bovine mastitis disease induces important economic losses all over the world. Nonetheless, the number of studies on the presence and role of viral agents in mastitis cases remains low. BPIV-3 and BoHV-1 are the most important agents among viral pathogens shown to be responsible for mastitis cases [7,28]. Furthermore, the severity of disease is increased by various factors, such as stress, environmental conditions, and immunodeficiency. This disease occurs in many mammalian species, especially domestic dairy cows [29,30]. In previous studies, the presence and role of bacterial agents in mastitis cases are emphasized [30]. 

The recognition of viral antigens by T cells is essential for host defense against viral infection. T cells can bind to major MHC class I or class II molecules that are antigenic proteins at the cell surface. While CD8 T cells recognize MHC class I molecules, CD4 T cells recognize MHC class II molecules. CD8 T cells are referred to as cytolytic T lymphocytes (CTLs), and can produce cytokines. CD4 T cells can produce some cytokines that are important for the synthesis of antibodies and for the proliferation of CD8 T cells. Thus, they are referred to as Th cells. MHC class I and class II molecules play a role in the different pathways, including cytosolic and endosomal pathways, respectively. Therefore, CD8 CTLs eradicate intracellular pathogens, whereas CD4 helper T cells help to eliminate extracellular pathogens with B cells and cytokines [31]. Our findings indicated that BPV3 and BoHV infection in cattle mammary tissues induced an immune response activation of the *CD4*+ and *CD8*+ T cells. In addition, the higher *CD4* gene expression level compared with that of *CD8* showed that the endosomal pathway may be more active in the immune response in the case of BPIV-3 and BoHV-1 infection. 

The IFNs, pro-inflammatory cytokines, were originally identified as cytokines that prevent viral replication. In addition, the IFN system provides the first response of immune defense against viral infections in vertebrates [32]. It can inhibit the viral infection by blocking virus replication and eliminating the virus-infected cells [33]. The type II family of IFNs includes a single member, interferon-γ (*IFN*-*γ*). IFN-γ has an effective role in innate and acquired immunity [34]. It has been reported that both *CD4* and *CD8* T cells are the primary sources of *IFN*-*γ* [35]. *IFN*-*γ* is an important cytokine that regulates several cellular processes through the transcriptional control of genes. Th1 cells that are essential for T cell-mediated immune response against intracellular infection are known to produce *IFN*-*γ*, *TGF*-β, and *IL*-2 cytokines, and are strongly associated with cell-mediated immunity inhibiting viral replication through the production of *IFN*-*γ* [35,36]. Th1 cytokines, including IL-2 and (*IFN*-*γ*), are strongly associated with directing cell-mediated immunity, such as by activation of macrophages [37]. In the present study, the increased *IFN-γ* expression level suggested that *IFN*-*γ* plays a role in the immune response against BPIV-3 and BoHV-1 infection in cattle mammary tissues. Furthermore, this result indicated that cell-mediated immunity is important in BPIV-3 and BoHV-1 infection. 

Previous studies have reported that *IFN*-*γ* treatment can decrease viral titers in infected neuronal cells compared with untreated cells. It demonstrates that *IFN*-*γ* has potent antiviral activity [38]. In other cases of virus infection, including herpes simplex virus type 1, *IFN*-*γ* has been shown to limit transsynaptic transmission, prevent viral reactivation, and inhibit virally induced apoptosis [39,40,41]. *CD8*+ and *CD4*+ T cells have been shown to contribute to *IFN*-*γ* expression during a number of viral infections [42,43]. The common hypothesis has been that *CD8*+ T cell responses play active a role in antiviral immunity. While this remark is right for several viruses, recent studies show that *CD4*+ T cells also play role in the antiviral response [44,45,46,47,48,49,50]. Recent studies, including one in this issue, have shown the critical role of *CD4* T cells in protection against viral infection [51]. The previous study has reported that *CD4*+ T cells are effectors for antiviral immunity due to secretion of key antiviral cytokines or direct cytotoxic effects [52]. The present study reports that in case of viral infection (BPIV-3 and BoHV-1), *CD4*, *CD8*, and *IFN*-*γ* gene expression levels were increased. This result indicated that *CD4*+ T cells, *CD8*+ T cells, and *IFN-γ* play a major role in antiviral immunity; this finding is supported by previous studies. 

Because the immune response to BoHV-1 is not identical to that of herpes simplex virus in mice or in humans, the immune response of cattle to BoHV-1 infection is unique and does not correlate precisely with experimental BoHV-1 infection in mice [53,54]. The limiting cell type for antigen-induced proliferation in BoHV-1 infection is *CD4*+ T [55]. *CD4*+ T lymphocytes form primary cytolytic *CD8*+ T cells against some HSV-1 antigens [56,57]. *CD8* T cell responses have been shown to be important mediators of immunity to a number of herpes virus infections. There is compelling evidence that *CD8+* cells are required in preventing the reactivation of latent infection and that they also contribute to the resolution of the acute phase of infection [58,59]. The prominent role of *CD8* T cells in immunity is also consistent with the capacity of herpes viruses, including BoHV-1, to inhibit a number of the steps involved in processing and presentation of antigen to *CD8* T cells [60,61], thus reducing the efficiency of the *CD8* T cell response [62]. Our findings revealed that BoHV-1 infection in mammary tissues causes the activation of *CD4* and *CD8*. 

While type I IFNs, especially *IFN*-α and *IFN*-β, are well-known in their antiviral role; some viruses restrict type I *IFN* production and responses. Previous studies have revealed that paramyxoviruses block type I *IFN* production and downstream signaling pathways [63,64]. A new class of *IFN*s, type III *IFN*s or *IFN*-λs, were described independently from two groups [65]. The recent study has reported that PIV-3 prevent the phosphorylation of Stat1 downstream of the type III *IFN* receptor [66]. *CD4*+ and *CD8*+ in PIV3 infected tissues produced Th1-polarized effector molecules, such as *IFN*-*γ*, *TNF*-α, and granzyme B, and they kill the antigen-loaded targets [67]. However, the effect of PIV-3 on the type II family of *IFN*s, *IFN*-*γ*, is unknown. In the present study, BPIV-3 infection increased the expression of IFN-*γ* in the mammary tissues of cattle. This result may be a new aspect for antiviral immunity against BPIV-3. 

## 4. Material and Method

### 4.1. Material

The materials used in this study consisted of 120 cattle mammary tissues obtained from the Oral Meat Entegre Facility in Erzurum. The mammary tissue samples were obtained from 120 multiparous (between parities two and eight), Holstein (n = 120) dairy cows with no signs of inflammation of the mammary glands as macroscopic. Collected samples were brought to the laboratory for total RNA isolation procedures, real-time PCR, and immunohistochemistry staining. 

### 4.2. Total RNA Isolation and cDNA Sythesis

Total RNA isolation was realized using TRIzol® (Invitrogen, Cat No: 15596026, USA). Mammary tissues (100 mg) were lysed and homogenized in 1mL Trizol reagent with a tissue homogenizer (Qiagen, TisueLyser II, Germany). Total RNA isolation process was performed in accordance with Trizol manufacturer procedure. The RNA concentration was measured using NanoDrop (Epoch Microplate Spectrophotometer, USA). The concentration of RNA samples was between 1250 ng/µL–1350 ng/µL. Total RNA samples quality were evaluated regarding DNA contamination by using gel electrophoresis. cDNA synthesis was performed using QuantiTect reverse transcription (Qiagen, Cat:330411 Germany). cDNA synthesis process was realized in accordance with kit’s manufacturer procedure. 

### 4.3. Real Time PCR

Real-time quantitative PCR (RT-qPCR) was performed to identify BPIV-3, BoHV-1 virus and the gene expression of *IFN*, *CD4*, and *CD8* in the mammary tissues with the CFX96 TouchTM Real-Time PCR Detection System (Bio-Rad, USA). *β-Actin* was used as a reference gene for RT-qPCR. Real-time PCR primers were designed in accordance with the sequence of cattle (*Bos Taurus*) using the primer design program Oligo 6.0 and Primer 5.0. *IFN, CD4*, and *CD8* primer sequences and reaction conditions were shown in Table 3. BPIV-3 and BoHV-1 primer sequences and reaction conditions were also shown in Table 3 and these primer sequences were received from previously conducted studies [68,69]. Real-Time PCR was performed in a volume of 25 µL containing 12.5 µL 2x QuaniTect SYBR Green PCR Master Mix (Qiagen, Cat: 330500, Germany), which contained HotStart DNA Taq polymerase, 0.75 µL (5 µM) each primer, 0.75 µL (100 ng/µL) cDNA as a template, and 10.25 µL RNase and DNase free water. The PCR amplification was confirmed by agarose gel electrophoresis and melting curve analysis. Relative folds of expressions were assessed with the 2^−ΔΔCT^ method [28]. The qRT-PCR assay was performed in accordance with the MIQE guidelines [70]. 

### 4.4. Immunohistochemistry Staining 

After the routine histopathology processing, all samples were poured into paraffin for blocking and prepared microtome sections in 5 μm. After the deparaffinization of the tissues on the polylysine slide, the plates were incubated in 3% H_2_O_2_ to inactivate the endogenous peroxidase activity and washed with PBS. Then the slides were immersed in antigen retrieval solution (ab 96674, abcam, USA) (pH 6.0) and heated in a microwave for 10 min to unmasked antigens. After cooling, sections were incubated for 10 min with a protein block solution (ab 80436, Abcam, USA) to prevent nonspecific binding. Sections were incubated for immunohistochemistry staining with primer antibodies (CD8 antibody: Mouse anti Bovine CD8 antibody, clone CC63, MCA837GA, Bio-Rad (Formerly AbD Serotec), CD 4 antibody: Mouse anti Bovine CD4 antibody, MCA834GA by Bio-Rad (Formerly AbD Serotec), IFN-γ antibody: Mouse anti Bovine Interferon Gamma Antibody, MCA1964 by Bio-Rad (Formerly AbD Serotec)) at room temperature for 30 minutes. After incubation, the procedure specified by EXPOSE Mouse and Rabbit Specific HRP/DAB Detection IHC Kit (ab80436) was followed. Sections were incubated with 3.3 diaminobenzidine (DAB) and then counterstained with Mayer’s hematoxylin (MH). The immunoreactivity observed with light microscopy was semi-quantitatively estimated using the following scale: absent, 0; low reactivity, 1; intermediate reactivity, 2; or high reactivity, 3.

### 4.5. Statistical Analysis

IBM SPSS 20 was used to realize the statistical analyses. One-way analysis of variance was (ANOVA) used to detect statistical differences of *IFGN*, *CD4*, and *CD8* expressions at mRNA transcript level between non-infected and infected mammary tissues. Relative mRNA fold change graphics were created by using the Graph pad prism software Inc., (Version 7.0, California, USA). qRT-PCR results are expressed as mean ± SEM (standard error of mean). Statistical differences were considered to be significant at *p* < 0.05, *p* < 0.01 and *p* < 0.001. For immunohistological, the differences among all groups were compared with nonparametric Kruskal–Wallis test. Dual cross-checks between the groups showing significant values were analyzed with a Mann–Whitney U-test (*p* < 0.05).

## 5. Conclusions

In conclusion, our findings indicated that natural BPIV-3 and BoHV-1 infections have a high prevalence in Bovine mammary tissues. This case revealed that significant precautions should be taken against viral agents in mastitis. In addition, it may be considered that the cellular immune response to viral infection is more active in BPIV-3 and BPIV-3 infected Bovine mammary tissues. Finally, this study demonstrates that IFN-γ is an important antiviral cytokine in BPIV-3 and BoHV-1 infections.

## Figures and Tables

**Table 1:** Real time RT-PCR results for 120 samples.

Material	BPIV-3	BoHV-1	BPIV-3 and BoHV-1
Macroscopic lesions	Absence	Absence	Absence
Mammary tissue	26	16	5
Percentage (%)	21.66%	13.3%	4.16%

**Table 2:** Immunoreactivity scale (mean + Std) of CD4+, CD8+ and IFN-γ in the mammarian tissue of five Bovine mammary tissues which is infected with BPIV-3 and BoHV-1. Means with the different letter, per each column, are significantly different according to Mann–Whitney U test, *p* < 0.05.

N = 5	CD4 + T cells	CD8 + T cells	IFN-γ
BPIV-3	0.80 ± 0.44 ^a^	2.20 ± 0.44 ^b^	1.40 ± 0.89 ^c^
BoHV-1	1.66 ± 0.51 ^aı^	1.00 ± 1.09 ^bı^	1.66 ± 1.03 ^cı^
BPIV-3 + BoHV-1	2.75 ± 0.50 ^aıı^	2.75 ± 0.50 ^aıı^	2.75 ± 0.50 ^cıı^

Means with the different letter and number per each column, are significantly different according to Mann–Whitney U test, *p* < 0.05.

**Table 3:** Pimer sequences of Beta Actin, IFN-γ, CD4, CD8, BPIV-3, and BoHV-1.

**Gene Name**	**Primer Sequence**	**Amplicon Size (bp)**	**Accession No**	**Reaction Conditions**
Bo-IFN-γ	F - AATTCCGGTGGATGATCTGC R - TCTCCGGCCTCGAAAGAGAT	130	NM_174086.1	94 °C 15 s/55 °C 30 s/72 °C 30 s (40 cycles)
Bo-CD4	F - TGATGAAAGTGACTAAGTCCCCA R- TTCGGCTGATTTGAGCCCTT	121	NM_001103225.1	94 °C 15 s/58 °C 30 s/72 °C 30 s (40 cycles)
Bo-CD8	F - CTGGACTTCGCCTGCAATATC R- CACGTCTTCGGTTCCGGC	112	NM_174015.1	94 °C 15 s/57 °C 30 s/72 °C 30 s (40 cycles)
Bo-Beta Actin	F - GTCGACACCGCAACCAGTTC R - TACGAGTCCTTCTGGCCCAT	181	AY141970.1	94 °C 15 s/57 °C 30 s/72 °C 30 s (40 cycles)
**Gene Name**	**Primer Sequences**	**Reference**	**Reactions Conditions**	
BPIV-3	F: TGATTGGATGTTCGGGAGTGA R: AGAATCCTTTCCTCAATCCTGATATACT	[28]	94 °C for 15 s, 58 °C for 30 s/72 °C for 30 s (40 cycle)	
BoHV-1	F: TGTGGACCTAAACCTCACGGT R: GTAGTCGAGCAGACCCGTGTC	[28]	94 °C for 15 s, 59 °C for 30 s/72 °C for 30 s (40 cycle)	

## Supplementary Materials

The following are available online at https://www.mdpi.com/2076-0817/8/1/26/s1, Figure S1: BPIV-3 and BoHV-1 nucleic acid signals, Figure S2: β-actin amplification signals for all tissues, Table S1: The Ct/Cq values of BoHV-1 and BPIV-3 for 120 samples.
