# Supplementary material for: Comparative Evaluation of the Immune Responses in Cattle Mammary Tissues Naturally Infected with Bovine Parainfluenza Virus Type 3 and Bovine Alphaherpesvirus-1
Source: Pathogens. 2019 Feb 25;8(1):26. doi: 10.3390/pathogens8010026 (PMC6470764; doi:10.3390/pathogens8010026)
Supplement: Supplementary file 1 [file pathogens-08-00026-s001.pdf]

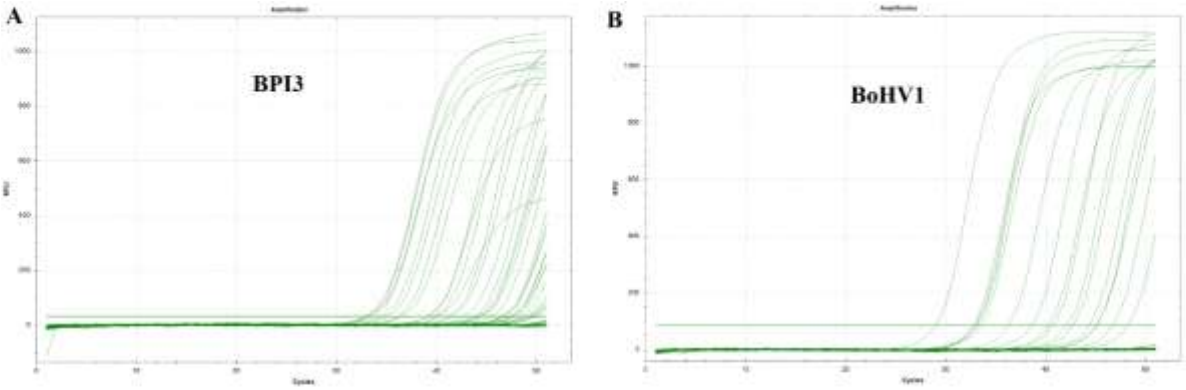

Figure S1. BPIV-3 and BoHV-1 nucleic acid signals. (A) BPIV-3. (B) BoHV-1.

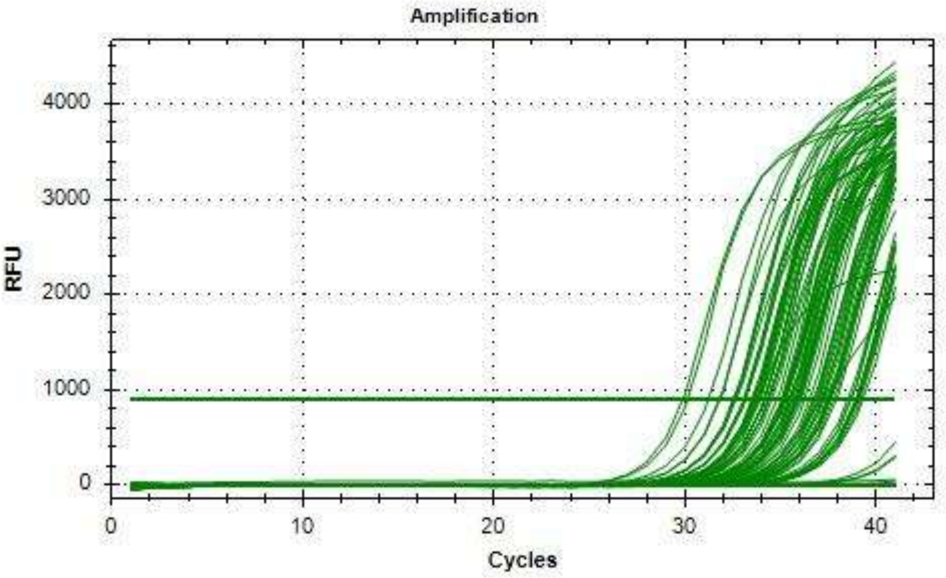

Figure S2. β-actin amplification signals for all tissues.

Table S1. The Ct/Cq values of BoHV-1 and BPIV-3 for 120 samples.

Sample	Cq BoHV-1	Cq BPIV-3
Sample 1	N/A	N/A
Sample 2	N/A	N/A
Sample 3	N/A	N/A
Sample 4	26.35	30.01
Sample 5	N/A	N/A
Sample 6	26.16	N/A
Sample 7	N/A	N/A
Sample 8	N/A	N/A
Sample 9	N/A	31.58
Sample 10	N/A	N/A
Sample 11	27.52	35.21
Sample 12	26.01	N/A
Sample 13	N/A	N/A
Sample 14	N/A	30.12

Sample 15	N/A	N/A
Sample 16	N/A	30.54
Sample 17	N/A	N/A
Sample 18	N/A	N/A
Sample 19	N/A	N/A
Sample 20	N/A	33.25
Sample 21	N/A	N/A
Sample 22	N/A	N/A
Sample 23	N/A	35.62
Sample 24	N/A	N/A
Sample 25	N/A	N/A
Sample 26	N/A	N/A
Sample 27	N/A	N/A
Sample 28	N/A	N/A
Sample 29	31.26	32.26
Sample 30	N/A	N/A
Sample 31	N/A	N/A
Sample 32	N/A	33.52
Sample 33	N/A	N/A
Sample 34	N/A	N/A
Sample 35	N/A	N/A
Sample 36	N/A	32.02
Sample 37	33.04	N/A
Sample 38	N/A	N/A
Sample 39	N/A	N/A
Sample 40	N/A	N/A
Sample 41	N/A	N/A
Sample 42	N/A	N/A
Sample 43	34.05	34.05
Sample 44	N/A	N/A
Sample 45	N/A	N/A
Sample 46	N/A	N/A
Sample 47	26.58	30.02
Sample 48	N/A	N/A
Sample 49	N/A	N/A
Sample 50	N/A	N/A
Sample 51	28.69	32.14
Sample 52	N/A	N/A
Sample 53	N/A	N/A
Sample 54	N/A	N/A
Sample 55	30.24	30.24
Sample 56	N/A	N/A
Sample 57	N/A	N/A
Sample 58	N/A	N/A
Sample 59	N/A	N/A
Sample 60	N/A	33.01
Sample 61	N/A	N/A
Sample 62	N/A	N/A
Sample 63	N/A	N/A
Sample 64	N/A	N/A

Sample 65	N/A	35.47
Sample 66	N/A	N/A
Sample 67	N/A	N/A
Sample 68	N/A	33.26
Sample 69	N/A	N/A
Sample 70	N/A	N/A
Sample 71	N/A	31.28
Sample 72	N/A	N/A
Sample 73	N/A	N/A
Sample 74	N/A	N/A
Sample 75	N/A	35.54
Sample 76	N/A	N/A
Sample 77	N/A	N/A
Sample 78	N/A	N/A
Sample 79	N/A	34.57
Sample 80	N/A	N/A
Sample 81	N/A	N/A
Sample 82	N/A	N/A
Sample 83	N/A	N/A
Sample 84	31.04	35.61
Sample 85	N/A	N/A
Sample 86	N/A	N/A
Sample 87	N/A	N/A
Sample 88	N/A	N/A
Sample 89	N/A	N/A
Sample 90	N/A	N/A
Sample 91	N/A	N/A
Sample 92	N/A	38.54
Sample 93	34.16	N/A
Sample 94	N/A	N/A
Sample 95	N/A	N/A
Sample 96	N/A	N/A
Sample 97	N/A	N/A
Sample 98	N/A	N/A
Sample 99	N/A	N/A
Sample 100	N/A	30.12
Sample 101	N/A	N/A
Sample 102	N/A	N/A
Sample 103	N/A	N/A
Sample 104	N/A	N/A
Sample 105	N/A	N/A
Sample 106	N/A	N/A
Sample 107	N/A	N/A
Sample 108	N/A	N/A
Sample 109	N/A	N/A
Sample 110	N/A	35.58
Sample 111	N/A	N/A
Sample 112	N/A	N/A
Sample 113	31.12	N/A
Sample 114	N/A	N/A

Sample 115	N/A	N/A
Sample 116	N/A	N/A
Sample 117	N/A	30.52
Sample 118	N/A	N/A
Sample 119	N/A	31.62
Sample 120	N/A	N/A
NTC	N/A	N/A
NRTC	N/A	N/A
NPR	N/A	N/A
